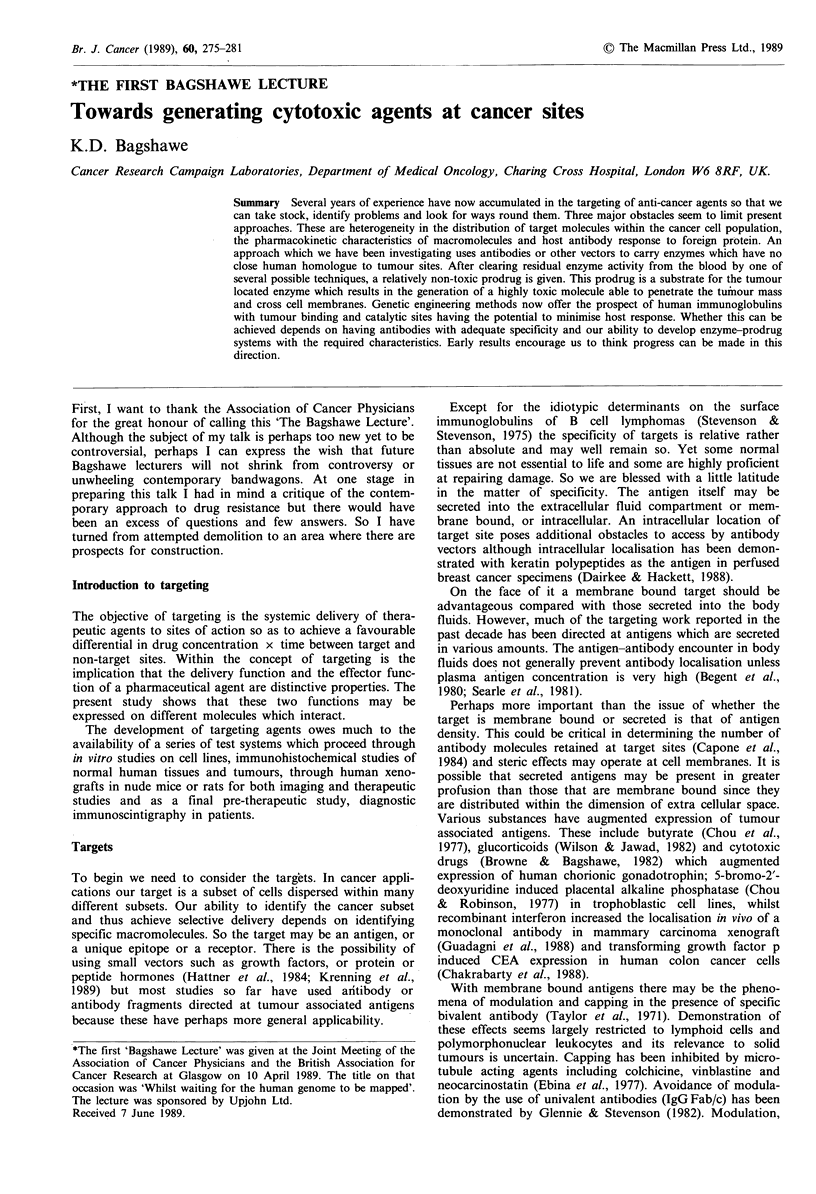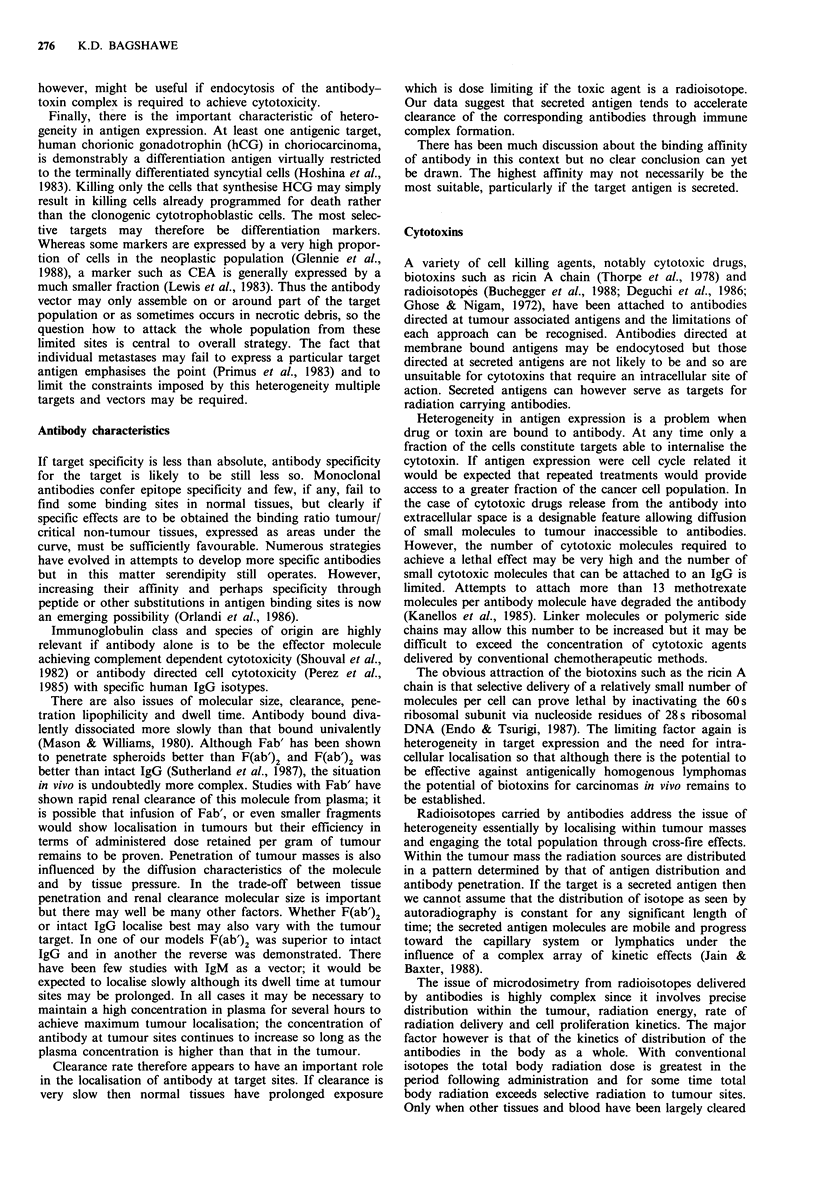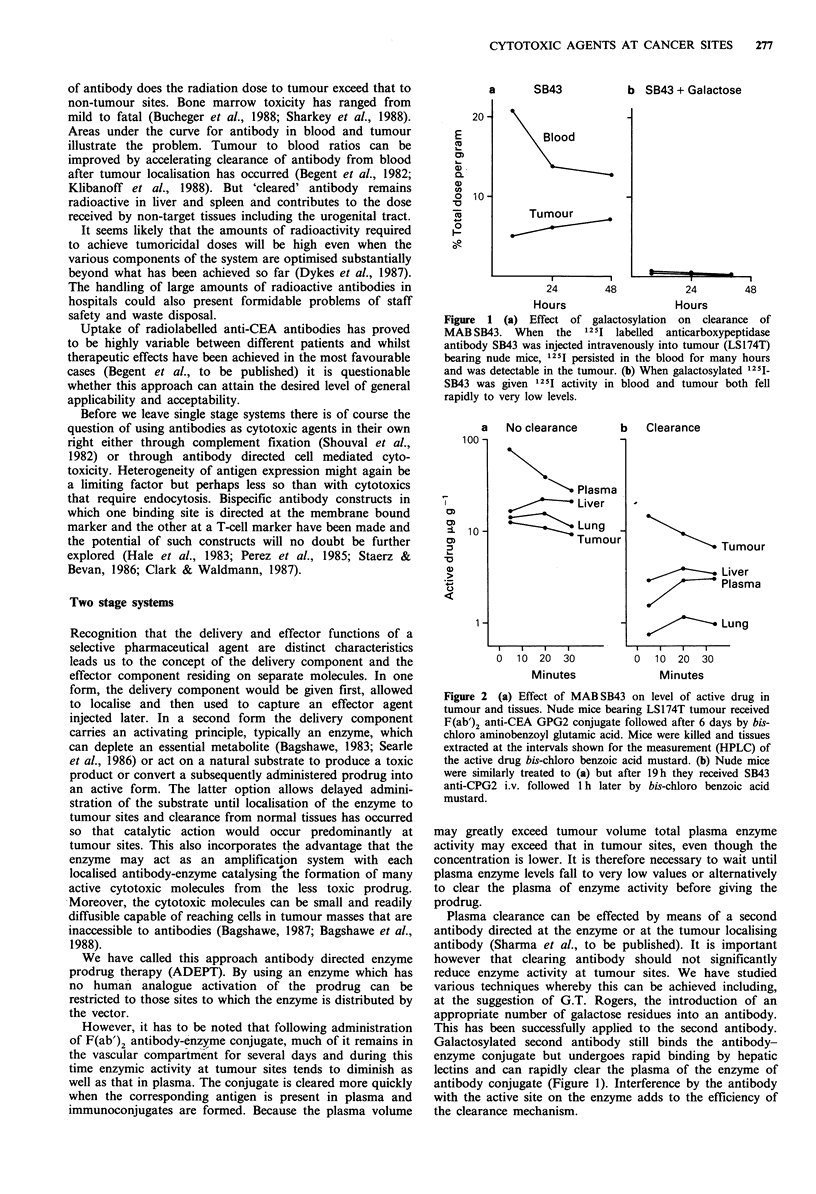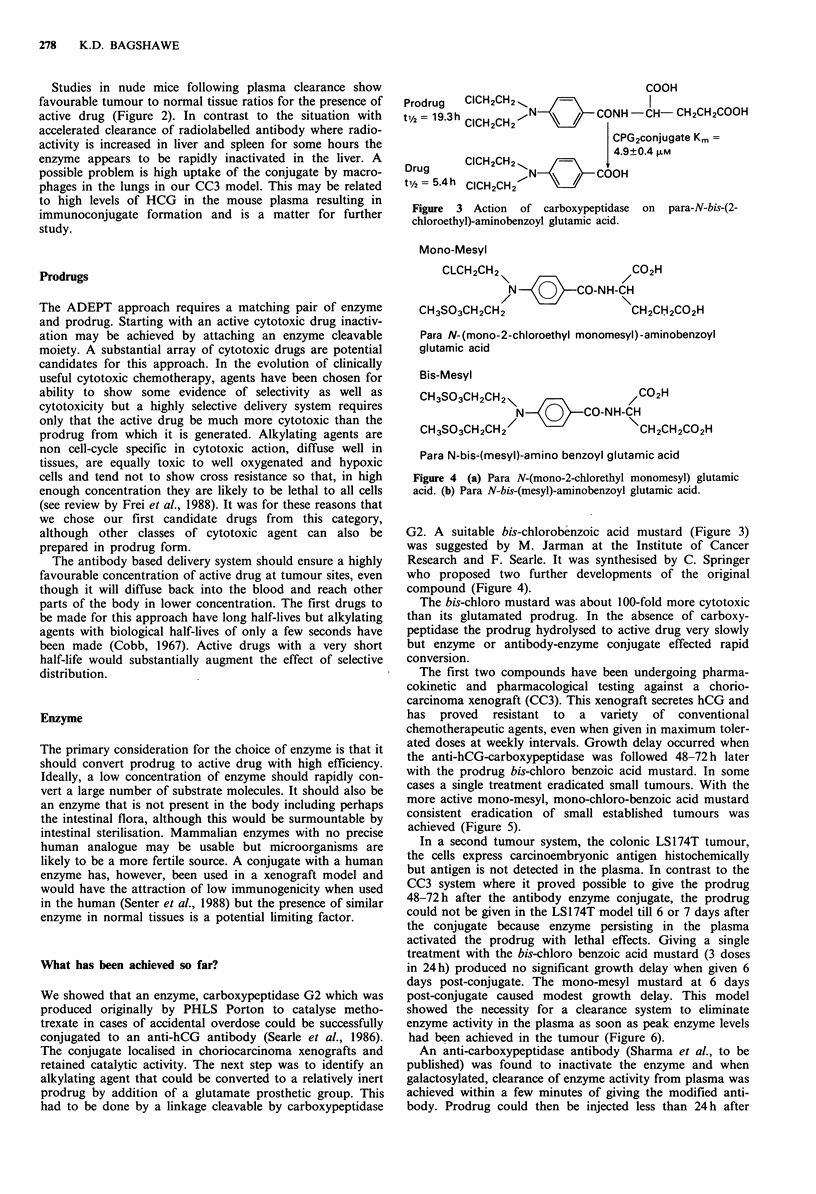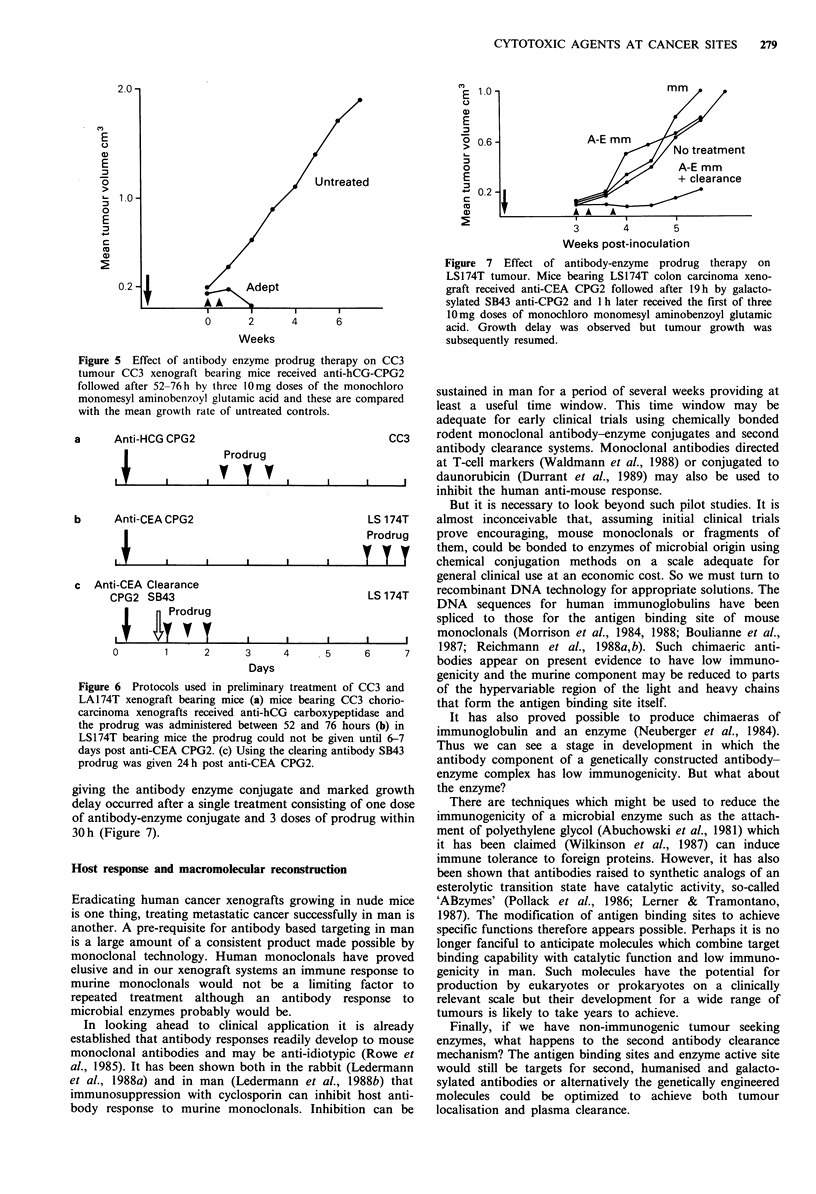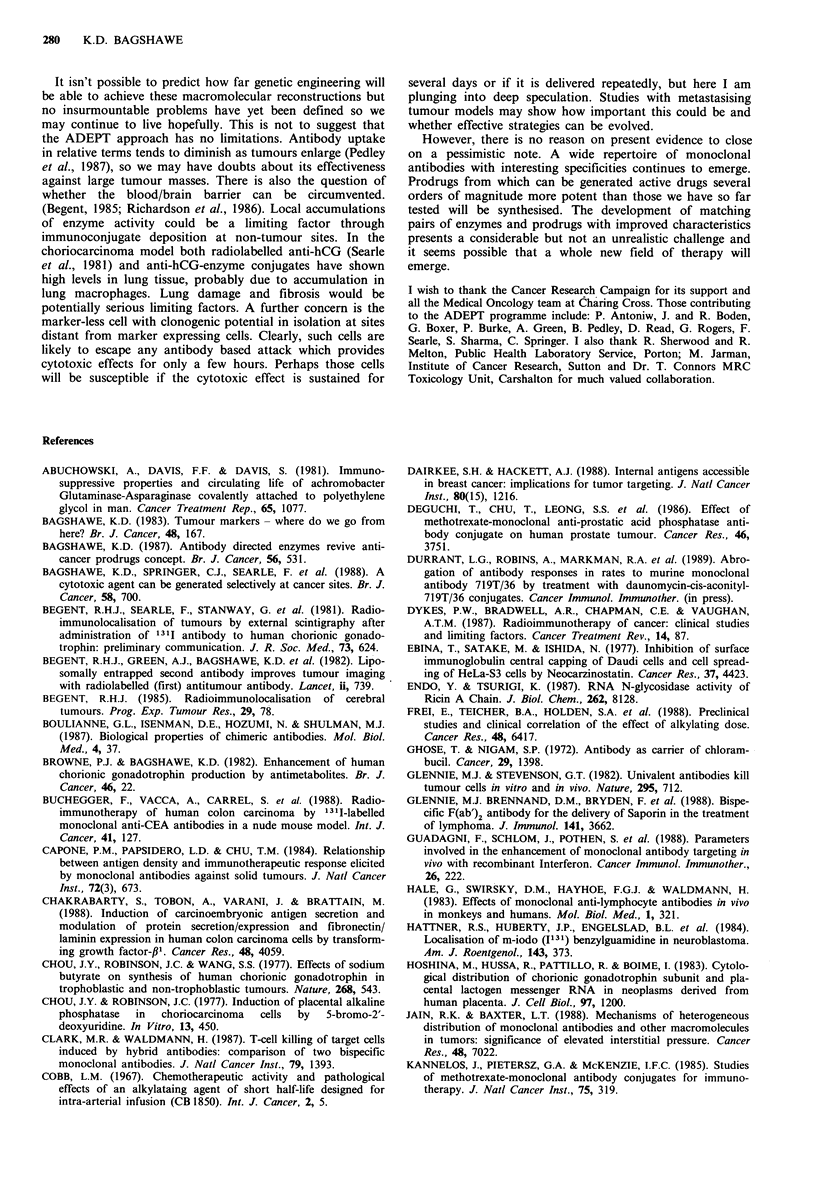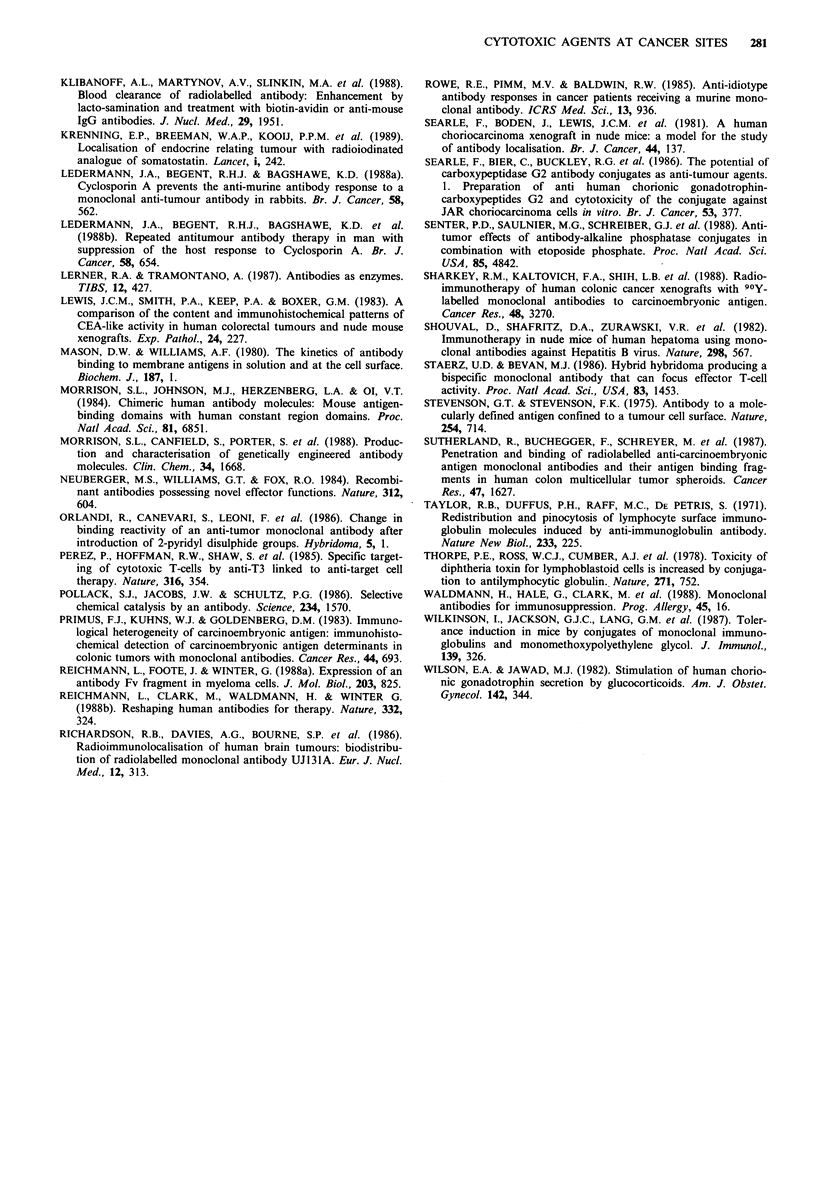# The First Bagshawe lecture. Towards generating cytotoxic agents at cancer sites.

**DOI:** 10.1038/bjc.1989.270

**Published:** 1989-09

**Authors:** K. D. Bagshawe

**Affiliations:** Department of Medical Oncology, Charing Cross Hospital, London, UK.

## Abstract

Several years of experience have now accumulated in the targeting of anti-cancer agents so that we can take stock, identify problems and look for ways round them. Three major obstacles seem to limit present approaches. These are heterogeneity in the distribution of target molecules within the cancer cell population, the pharmacokinetic characteristics of macromolecules and host antibody response to foreign protein. An approach which we have been investigating uses antibodies or other vectors to carry enzymes which have no close human homologue to tumour sites. After clearing residual enzyme activity from the blood by one of several possible techniques, a relatively non-toxic prodrug is given. This prodrug is a substrate for the tumour located enzyme which results in the generation of a highly toxic molecule able to penetrate the tumour mass and cross cell membranes. Genetic engineering methods now offer the prospect of human immunoglobulins with tumour binding and catalytic sites having the potential to minimise host response. Whether this can be achieved depends on having antibodies with adequate specificity and our ability to develop enzyme-prodrug systems with the required characteristics. Early results encourage us to think progress can be made in this direction.


*Br. J. Cancer* (1989), **60**, 275–281 © The Macmillan Press Ltd., 1989

### *THE FIRST BAGSHAWE LECTURE

### Towards generating cytotoxic agents at cancer sites

K.D. Bagshawe

*Cancer Research Campaign Laboratories, Department of Medical Oncology, Charing Cross Hospital, London W6 8RF, UK.*

**Summary** Several years of experience have now accumulated in the targeting of anti-cancer agents so that we can take stock, identify problems and look for ways round them. Three major obstacles seem to limit present approaches. These are heterogeneity in the distribution of target molecules within the cancer cell population, the pharmacokinetic characteristics of macromolecules and host antibody response to foreign protein. An approach which we have been investigating uses antibodies or other vectors to carry enzymes which have no close human homologue to tumour sites. After clearing residual enzyme activity from the blood by one of several possible techniques, a relatively non-toxic prodrug is given. This prodrug is a substrate for the tumour located enzyme which results in the generation of a highly toxic molecule able to penetrate the tumour mass and cross cell membranes. Genetic engineering methods now offer the prospect of human immunoglobulins with tumour binding and catalytic sites having the potential to minimise host response. Whether this can be achieved depends on having antibodies with adequate specificity and our ability to develop enzyme–prodrug systems with the required characteristics. Early results encourage us to think progress can be made in this direction.

First, I want to thank the Association of Cancer Physicians for the great honour of calling this 'The Bagshawe Lecture'. Although the subject of my talk is perhaps too new yet to be controversial, perhaps I can express the wish that future Bagshawe lecturers will not shrink from controversy or unwheeling contemporary bandwagons. At one stage in preparing this talk I had in mind a critique of the contemporary approach to drug resistance but there would have been an excess of questions and few answers. So I have turned from attempted demolition to an area where there are prospects for construction.

### Introduction to targeting

The objective of targeting is the systemic delivery of therapeutic agents to sites of action so as to achieve a favourable differential in drug concentration × time between target and non-target sites. Within the concept of targeting is the implication that the delivery function and the effector function of a pharmaceutical agent are distinctive properties. The present study shows that these two functions may be expressed on different molecules which interact.

The development of targeting agents owes much to the availability of a series of test systems which proceed through *in vitro* studies on cell lines, immunohistochemical studies of normal human tissues and tumours, through human xenografts in nude mice or rats for both imaging and therapeutic studies and as a final pre-therapeutic study, diagnostic immunoscintigraphy in patients.

### Targets

To begin we need to consider the targets. In cancer applications our target is a subset of cells dispersed within many different subsets. Our ability to identify the cancer subset and thus achieve selective delivery depends on identifying specific macromolecules. So the target may be an antigen, or a unique epitope or a receptor. There is the possibility of using small vectors such as growth factors, or protein or peptide hormones (Hattner *et al.*, 1984; Krenning *et al.*, 1989) but most studies so far have used antibody or antibody fragments directed at tumour associated antigens because these have perhaps more general applicability.

*The first 'Bagshawe Lecture' was given at the Joint Meeting of the Association of Cancer Physicians and the British Association for Cancer Research at Glasgow on 10 April 1989. The title on that occasion was 'Whilst waiting for the human genome to be mapped'. The lecture was sponsored by Upjohn Ltd.

Received 7 June 1989.

Except for the idiotypic determinants on the surface immunoglobulins of B cell lymphomas (Stevenson & Stevenson, 1975) the specificity of targets is relative rather than absolute and may well remain so. Yet some normal tissues are not essential to life and some are highly proficient at repairing damage. So we are blessed with a little latitude in the matter of specificity. The antigen itself may be secreted into the extracellular fluid compartment or membrane bound, or intracellular. An intracellular location of target site poses additional obstacles to access by antibody vectors although intracellular localisation has been demonstrated with keratin polypeptides as the antigen in perfused breast cancer specimens (Dairkee & Hackett, 1988).

On the face of it a membrane bound target should be advantageous compared with those secreted into the body fluids. However, much of the targeting work reported in the past decade has been directed at antigens which are secreted in various amounts. The antigen–antibody encounter in body fluids does not generally prevent antibody localisation unless plasma antigen concentration is very high (Begent *et al.*, 1980; Searle *et al.*, 1981).

Perhaps more important than the issue of whether the target is membrane bound or secreted is that of antigen density. This could be critical in determining the number of antibody molecules retained at target sites (Capone *et al.*, 1984) and steric effects may operate at cell membranes. It is possible that secreted antigens may be present in greater profusion than those that are membrane bound since they are distributed within the dimension of extra cellular space. Various substances have augmented expression of tumour associated antigens. These include butyrate (Chou *et al.*, 1977), glucorticoids (Wilson & Jawad, 1982) and cytotoxic drugs (Browne & Bagshawe, 1982) which augmented expression of human chorionic gonadotrophin; 5-bromo-2′-deoxyuridine induced placental alkaline phosphatase (Chou & Robinson, 1977) in trophoblastic cell lines, whilst recombinant interferon increased the localisation *in vivo* of a monoclonal antibody in mammary carcinoma xenograft (Guadagni *et al.*, 1988) and transforming growth factor p induced CEA expression in human colon cancer cells (Chakrabarty *et al.*, 1988).

With membrane bound antigens there may be the phenomena of modulation and capping in the presence of specific bivalent antibody (Taylor *et al.*, 1971). Demonstration of these effects seems largely restricted to lymphoid cells and polymorphonuclear leukocytes and its relevance to solid tumours is uncertain. Capping has been inhibited by microtubule acting agents including colchicine, vinblastine and neocarcinostatin (Ebina *et al.*, 1977). Avoidance of modulation by the use of univalent antibodies (IgG Fab/c) has been demonstrated by Glennie & Stevenson (1982). Modulation, however, might be useful if endocytosis of the antibody–toxin complex is required to achieve cytotoxicity.

Finally, there is the important characteristic of heterogeneity in antigen expression. At least one antigenic target, human chorionic gonadotrophin (hCG) in choriocarcinoma, is demonstrably a differentiation antigen virtually restricted to the terminally differentiated syncytial cells (Hoshina *et al.*, 1983). Killing only the cells that synthesise HCG may simply result in killing cells already programmed for death rather than the clonogenic cytotrophoblastic cells. The most selective targets may therefore be differentiation markers. Whereas some markers are expressed by a very high proportion of cells in the neoplastic population (Glennie *et al.*, 1988), a marker such as CEA is generally expressed by a much smaller fraction (Lewis *et al.*, 1983). Thus the antibody vector may only assemble on or around part of the target population or as sometimes occurs in necrotic debris, so the question how to attack the whole population from these limited sites is central to overall strategy. The fact that individual metastases may fail to express a particular target antigen emphasises the point (Primus *et al.*, 1983) and to limit the constraints imposed by this heterogeneity multiple targets and vectors may be required.

### Antibody characteristics

If target specificity is less than absolute, antibody specificity for the target is likely to be still less so. Monoclonal antibodies confer epitope specificity and few, if any, fail to find some binding sites in normal tissues, but clearly if specific effects are to be obtained the binding ratio tumour/critical non-tumour tissues, expressed as areas under the curve, must be sufficiently favourable. Numerous strategies have evolved in attempts to develop more specific antibodies but in this matter serendipity still operates. However, increasing their affinity and perhaps specificity through peptide or other substitutions in antigen binding sites is now an emerging possibility (Orlandi *et al.*, 1986).

Immunoglobulin class and species of origin are highly relevant if antibody alone is to be the effector molecule achieving complement dependent cytotoxicity (Shouval *et al.*, 1982) or antibody directed cell cytotoxicity (Perez *et al.*, 1985) with specific human IgG isotypes.

There are also issues of molecular size, clearance, penetration lipophilicity and dwell time. Antibody bound divalently dissociated more slowly than that bound univalently (Mason & Williams, 1980). Although Fab′ has been shown to penetrate spheroids better than $F(ab')_2$ and $F(ab')_2$ was better than intact IgG (Sutherland *et al.*, 1987), the situation *in vivo* is undoubtedly more complex. Studies with Fab′ have shown rapid renal clearance of this molecule from plasma; it is possible that infusion of Fab′, or even smaller fragments would show localisation in tumours but their efficiency in terms of administered dose retained per gram of tumour remains to be proven. Penetration of tumour masses is also influenced by the diffusion characteristics of the molecule and by tissue pressure. In the trade-off between tissue penetration and renal clearance molecular size is important but there may well be many other factors. Whether $F(ab')_2$ or intact IgG localise best may also vary with the tumour target. In one of our models $F(ab')_2$ was superior to intact IgG and in another the reverse was demonstrated. There have been few studies with IgM as a vector; it would be expected to localise slowly although its dwell time at tumour sites may be prolonged. In all cases it may be necessary to maintain a high concentration in plasma for several hours to achieve maximum tumour localisation; the concentration of antibody at tumour sites continues to increase so long as the plasma concentration is higher than that in the tumour.

Clearance rate therefore appears to have an important role in the localisation of antibody at target sites. If clearance is very slow then normal tissues have prolonged exposure which is dose limiting if the toxic agent is a radioisotope. Our data suggest that secreted antigen tends to accelerate clearance of the corresponding antibodies through immune complex formation.

There has been much discussion about the binding affinity of antibody in this context but no clear conclusion can yet be drawn. The highest affinity may not necessarily be the most suitable, particularly if the target antigen is secreted.

### Cytotoxins

A variety of cell killing agents, notably cytotoxic drugs, biotoxins such as ricin A chain (Thorpe *et al.*, 1978) and radioisotopes (Buchegger *et al.*, 1988; Deguchi *et al.*, 1986; Ghose & Nigam, 1972), have been attached to antibodies directed at tumour associated antigens and the limitations of each approach can be recognised. Antibodies directed at membrane bound antigens may be endocytosed but those directed at secreted antigens are not likely to be and so are unsuitable for cytotoxins that require an intracellular site of action. Secreted antigens can however serve as targets for radiation carrying antibodies.

Heterogeneity in antigen expression is a problem when drug or toxin are bound to antibody. At any time only a fraction of the cells constitute targets able to internalise the cytotoxin. If antigen expression were cell cycle related it would be expected that repeated treatments would provide access to a greater fraction of the cancer cell population. In the case of cytotoxic drugs release from the antibody into extracellular space is a designable feature allowing diffusion of small molecules to tumour inaccessible to antibodies. However, the number of cytotoxic molecules required to achieve a lethal effect may be very high and the number of small cytotoxic molecules that can be attached to an IgG is limited. Attempts to attach more than 13 methotrexate molecules per antibody molecule have degraded the antibody (Kanellos *et al.*, 1985). Linker molecules or polymeric side chains may allow this number to be increased but it may be difficult to exceed the concentration of cytotoxic agents delivered by conventional chemotherapeutic methods.

The obvious attraction of the biotoxins such as the ricin A chain is that selective delivery of a relatively small number of molecules per cell can prove lethal by inactivating the 60 s ribosomal subunit via nucleoside residues of 28 s ribosomal DNA (Endo & Tsurigi, 1987). The limiting factor again is heterogeneity in target expression and the need for intracellular localisation so that although there is the potential to be effective against antigenically homogenous lymphomas the potential of biotoxins for carcinomas *in vivo* remains to be established.

Radioisotopes carried by antibodies address the issue of heterogeneity essentially by localising within tumour masses and engaging the total population through cross-fire effects. Within the tumour mass the radiation sources are distributed in a pattern determined by that of antigen distribution and antibody penetration. If the target is a secreted antigen then we cannot assume that the distribution of isotope as seen by autoradiography is constant for any significant length of time; the secreted antigen molecules are mobile and progress toward the capillary system or lymphatics under the influence of a complex array of kinetic effects (Jain & Baxter, 1988).

The issue of microdosimetry from radioisotopes delivered by antibodies is highly complex since it involves precise distribution within the tumour, radiation energy, rate of radiation delivery and cell proliferation kinetics. The major factor however is that of the kinetics of distribution of the antibodies in the body as a whole. With conventional isotopes the total body radiation dose is greatest in the period following administration and for some time total body radiation exceeds selective radiation to tumour sites. Only when other tissues and blood have been largely cleared of antibody does the radiation dose to tumour exceed that to non-tumour sites. Bone marrow toxicity has ranged from mild to fatal (Buchegar *et al.*, 1988; Sharkey *et al.*, 1988). Areas under the curve for antibody in blood and tumour illustrate the problem. Tumour to blood ratios can be improved by accelerating clearance of antibody from blood after tumour localisation has occurred (Begent *et al.*, 1982; Klibanoff *et al.*, 1988). But 'cleared' antibody remains radioactive in liver and spleen and contributes to the dose received by non-target tissues including the urogenital tract.

It seems likely that the amounts of radioactivity required to achieve tumoricidal doses will be high even when the various components of the system are optimised substantially beyond what has been achieved so far (Dykes *et al.*, 1987). The handling of large amounts of radioactive antibodies in hospitals could also present formidable problems of staff safety and waste disposal.

Uptake of radiolabelled anti-CEA antibodies has proved to be highly variable between different patients and whilst therapeutic effects have been achieved in the most favourable cases (Begent *et al.*, to be published) it is questionable whether this approach can attain the desired level of general applicability and acceptability.

Before we leave single stage systems there is of course the question of using antibodies as cytotoxic agents in their own right either through complement fixation (Shouval *et al.*, 1982) or through antibody directed cell mediated cytotoxicity. Heterogeneity of antigen expression might again be a limiting factor but perhaps less so than with cytotoxics that require endocytosis. Bispecific antibody constructs in which one binding site is directed at the membrane bound marker and the other at a T-cell marker have been made and the potential of such constructs will no doubt be further explored (Hale *et al.*, 1983; Perez *et al.*, 1985; Staerz & Bevan, 1986; Clark & Waldmann, 1987).

### Two stage systems

Recognition that the delivery and effector functions of a selective pharmaceutical agent are distinct characteristics leads us to the concept of the delivery component and the effector component residing on separate molecules. In one form, the delivery component would be given first, allowed to localise and then used to capture an effector agent injected later. In a second form the delivery component carries an activating principle, typically an enzyme, which can deplete an essential metabolite (Bagshawe, 1983; Searle *et al.*, 1986) or act on a natural substrate to produce a toxic product or convert a subsequently administered prodrug into an active form. The latter option allows delayed administration of the substrate until localisation of the enzyme to tumour sites and clearance from normal tissues has occurred so that catalytic action would occur predominantly at tumour sites. This also incorporates the advantage that the enzyme may act as an amplification system with each localised antibody-enzyme catalysing the formation of many active cytotoxic molecules from the less toxic prodrug. Moreover, the cytotoxic molecules can be small and readily diffusible capable of reaching cells in tumour masses that are inaccessible to antibodies (Bagshawe, 1987; Bagshawe *et al.*, 1988).

We have called this approach antibody directed enzyme prodrug therapy (ADEPT). By using an enzyme which has no human analogue activation of the prodrug can be restricted to those sites to which the enzyme is distributed by the vector.

However, it has to be noted that following administration of $F(ab')_2$ antibody-enzyme conjugate, much of it remains in the vascular compartment for several days and during this time enzymic activity at tumour sites tends to diminish as well as that in plasma. The conjugate is cleared more quickly when the corresponding antigen is present in plasma and immunoconjugates are formed. Because the plasma volume

**Figure 1** (**a**) Effect of galactosylation on clearance of MAB SB43. When the $^{125}I$ labelled anticarboxypeptidase antibody SB43 was injected intravenously into tumour (LS174T) bearing nude mice, $^{125}I$ persisted in the blood for many hours and was detectable in the tumour. (**b**) When galactosylated $^{125}I$-SB43 was given $^{125}I$ activity in blood and tumour both fell rapidly to very low levels.

**Figure 2** (**a**) Effect of MAB SB43 on level of active drug in tumour and tissues. Nude mice bearing LS174T tumour received $F(ab')_2$ anti-CEA GPG2 conjugate followed after 6 days by *bis*-chloro aminobenzoyl glutamic acid. Mice were killed and tissues extracted at the intervals shown for the measurement (HPLC) of the active drug *bis*-chloro benzoic acid mustard. (**b**) Nude mice were similarly treated to (**a**) but after 19 h they received SB43 anti-CPG2 i.v. followed 1 h later by *bis*-chloro benzoic acid mustard.

may greatly exceed tumour volume total plasma enzyme activity may exceed that in tumour sites, even though the concentration is lower. It is therefore necessary to wait until plasma enzyme levels fall to very low values or alternatively to clear the plasma of enzyme activity before giving the prodrug.

Plasma clearance can be effected by means of a second antibody directed at the enzyme or at the tumour localising antibody (Sharma *et al.*, to be published). It is important however that clearing antibody should not significantly reduce enzyme activity at tumour sites. We have studied various techniques whereby this can be achieved including, at the suggestion of G.T. Rogers, the introduction of an appropriate number of galactose residues into an antibody. This has been successfully applied to the second antibody. Galactosylated second antibody still binds the antibody–enzyme conjugate but undergoes rapid binding by hepatic lectins and can rapidly clear the plasma of the enzyme of antibody conjugate (Figure 1). Interference by the antibody with the active site on the enzyme adds to the efficiency of the clearance mechanism.

Studies in nude mice following plasma clearance show favourable tumour to normal tissue ratios for the presence of active drug (Figure 2). In contrast to the situation with accelerated clearance of radiolabelled antibody where radioactivity is increased in liver and spleen for some hours the enzyme appears to be rapidly inactivated in the liver. A possible problem is high uptake of the conjugate by macrophages in the lungs in our CC3 model. This may be related to high levels of HCG in the mouse plasma resulting in immunoconjugate formation and is a matter for further study.

### Prodrugs

The ADEPT approach requires a matching pair of enzyme and prodrug. Starting with an active cytotoxic drug inactivation may be achieved by attaching an enzyme cleavable moiety. A substantial array of cytotoxic drugs are potential candidates for this approach. In the evolution of clinically useful cytotoxic chemotherapy, agents have been chosen for ability to show some evidence of selectivity as well as cytotoxicity but a highly selective delivery system requires only that the active drug be much more cytotoxic than the prodrug from which it is generated. Alkylating agents are non cell-cycle specific in cytotoxic action, diffuse well in tissues, are equally toxic to well oxygenated and hypoxic cells and tend not to show cross resistance so that, in high enough concentration they are likely to be lethal to all cells (see review by Frei *et al.*, 1988). It was for these reasons that we chose our first candidate drugs from this category, although other classes of cytotoxic agent can also be prepared in prodrug form.

The antibody based delivery system should ensure a highly favourable concentration of active drug at tumour sites, even though it will diffuse back into the blood and reach other parts of the body in lower concentration. The first drugs to be made for this approach have long half-lives but alkylating agents with biological half-lives of only a few seconds have been made (Cobb, 1967). Active drugs with a very short half-life would substantially augment the effect of selective distribution.

### Enzyme

The primary consideration for the choice of enzyme is that it should convert prodrug to active drug with high efficiency. Ideally, a low concentration of enzyme should rapidly convert a large number of substrate molecules. It should also be an enzyme that is not present in the body including perhaps the intestinal flora, although this would be surmountable by intestinal sterilisation. Mammalian enzymes with no precise human analogue may be usable but microorganisms are likely to be a more fertile source. A conjugate with a human enzyme has, however, been used in a xenograft model and would have the attraction of low immunogenicity when used in the human (Senter *et al.*, 1988) but the presence of similar enzyme in normal tissues is a potential limiting factor.

### What has been achieved so far?

We showed that an enzyme, carboxypeptidase G2 which was produced originally by PHLS Porton to catalyse methotrexate in cases of accidental overdose could be successfully conjugated to an anti-hCG antibody (Searle *et al.*, 1986). The conjugate localised in choriocarcinoma xenografts and retained catalytic activity. The next step was to identify an alkylating agent that could be converted to a relatively inert prodrug by addition of a glutamate prosthetic group. This had to be done by a linkage cleavable by carboxypeptidase

**Figure 3** Action of carboxypeptidase on para-*N-bis*-(2-chloroethyl)-aminobenzoyl glutamic acid.

**Figure 4** **(a)** Para *N*-(mono-2-chlorethyl monomesyl) glutamic acid. **(b)** Para *N-bis*-(mesyl)-aminobenzoyl glutamic acid.

G2. A suitable *bis*-chlorobenzoic acid mustard (Figure 3) was suggested by M. Jarman at the Institute of Cancer Research and F. Searle. It was synthesised by C. Springer who proposed two further developments of the original compound (Figure 4).

The *bis*-chloro mustard was about 100-fold more cytotoxic than its glutamated prodrug. In the absence of carboxypeptidase the prodrug hydrolysed to active drug very slowly but enzyme or antibody-enzyme conjugate effected rapid conversion.

The first two compounds have been undergoing pharmacokinetic and pharmacological testing against a choriocarcinoma xenograft (CC3). This xenograft secretes hCG and has proved resistant to a variety of conventional chemotherapeutic agents, even when given in maximum tolerated doses at weekly intervals. Growth delay occurred when the anti-hCG-carboxypeptidase was followed 48–72 h later with the prodrug *bis*-chloro benzoic acid mustard. In some cases a single treatment eradicated small tumours. With the more active mono-mesyl, mono-chloro-benzoic acid mustard consistent eradication of small established tumours was achieved (Figure 5).

In a second tumour system, the colonic LS174T tumour, the cells express carcinoembryonic antigen histochemically but antigen is not detected in the plasma. In contrast to the CC3 system where it proved possible to give the prodrug 48–72 h after the antibody enzyme conjugate, the prodrug could not be given in the LS174T model till 6 or 7 days after the conjugate because enzyme persisting in the plasma activated the prodrug with lethal effects. Giving a single treatment with the *bis*-chloro benzoic acid mustard (3 doses in 24 h) produced no significant growth delay when given 6 days post-conjugate. The mono-mesyl mustard at 6 days post-conjugate caused modest growth delay. This model showed the necessity for a clearance system to eliminate enzyme activity in the plasma as soon as peak enzyme levels had been achieved in the tumour (Figure 6).

An anti-carboxypeptidase antibody (Sharma *et al.*, to be published) was found to inactivate the enzyme and when galactosylated, clearance of enzyme activity from plasma was achieved within a few minutes of giving the modified antibody. Prodrug could then be injected less than 24 h after

**Figure 5** Effect of antibody enzyme prodrug therapy on CC3 tumour CC3 xenograft bearing mice received anti-hCG-CPG2 followed after 52–76 h by three 10 mg doses of the monochloro monomesyl aminobenzoyl glutamic acid and these are compared with the mean growth rate of untreated controls.

**Figure 6** Protocols used in preliminary treatment of CC3 and LA174T xenograft bearing mice (**a**) mice bearing CC3 choriocarcinoma xenografts received anti-hCG carboxypeptidase and the prodrug was administered between 52 and 76 hours (**b**) in LS174T bearing mice the prodrug could not be given until 6–7 days post anti-CEA CPG2. (c) Using the clearing antibody SB43 prodrug was given 24 h post anti-CEA CPG2.

giving the antibody enzyme conjugate and marked growth delay occurred after a single treatment consisting of one dose of antibody-enzyme conjugate and 3 doses of prodrug within 30 h (Figure 7).

### Host response and macromolecular reconstruction

Eradicating human cancer xenografts growing in nude mice is one thing, treating metastatic cancer successfully in man is another. A pre-requisite for antibody based targeting in man is a large amount of a consistent product made possible by monoclonal technology. Human monoclonals have proved elusive and in our xenograft systems an immune response to murine monoclonals would not be a limiting factor to repeated treatment although an antibody response to microbial enzymes probably would be.

In looking ahead to clinical application it is already established that antibody responses readily develop to mouse monoclonal antibodies and may be anti-idiotypic (Rowe *et al.*, 1985). It has been shown both in the rabbit (Ledermann *et al.*, 1988*a*) and in man (Ledermann *et al.*, 1988*b*) that immunosuppression with cyclosporin can inhibit host antibody response to murine monoclonals. Inhibition can be

**Figure 7** Effect of antibody-enzyme prodrug therapy on LS174T tumour. Mice bearing LS174T colon carcinoma xenograft received anti-CEA CPG2 followed after 19 h by galactosylated SB43 anti-CPG2 and 1 h later received the first of three 10 mg doses of monochloro monomesyl aminobenzoyl glutamic acid. Growth delay was observed but tumour growth was subsequently resumed.

sustained in man for a period of several weeks providing at least a useful time window. This time window may be adequate for early clinical trials using chemically bonded rodent monoclonal antibody–enzyme conjugates and second antibody clearance systems. Monoclonal antibodies directed at T-cell markers (Waldmann *et al.*, 1988) or conjugated to daunorubicin (Durrant *et al.*, 1989) may also be used to inhibit the human anti-mouse response.

But it is necessary to look beyond such pilot studies. It is almost inconceivable that, assuming initial clinical trials prove encouraging, mouse monoclonals or fragments of them, could be bonded to enzymes of microbial origin using chemical conjugation methods on a scale adequate for general clinical use at an economic cost. So we must turn to recombinant DNA technology for appropriate solutions. The DNA sequences for human immunoglobulins have been spliced to those for the antigen binding site of mouse monoclonals (Morrison *et al.*, 1984, 1988; Boulianne *et al.*, 1987; Reichmann *et al.*, 1988*a*,*b*). Such chimaeric antibodies appear on present evidence to have low immunogenicity and the murine component may be reduced to parts of the hypervariable region of the light and heavy chains that form the antigen binding site itself.

It has also proved possible to produce chimaeras of immunoglobulin and an enzyme (Neuberger *et al.*, 1984). Thus we can see a stage in development in which the antibody component of a genetically constructed antibody–enzyme complex has low immunogenicity. But what about the enzyme?

There are techniques which might be used to reduce the immunogenicity of a microbial enzyme such as the attachment of polyethylene glycol (Abuchowski *et al.*, 1981) which it has been claimed (Wilkinson *et al.*, 1987) can induce immune tolerance to foreign proteins. However, it has also been shown that antibodies raised to synthetic analogs of an esterolytic transition state have catalytic activity, so-called 'ABzymes' (Pollack *et al.*, 1986; Lerner & Tramontano, 1987). The modification of antigen binding sites to achieve specific functions therefore appears possible. Perhaps it is no longer fanciful to anticipate molecules which combine target binding capability with catalytic function and low immunogenicity in man. Such molecules have the potential for production by eukaryotes or prokaryotes on a clinically relevant scale but their development for a wide range of tumours is likely to take years to achieve.

Finally, if we have non-immunogenic tumour seeking enzymes, what happens to the second antibody clearance mechanism? The antigen binding sites and enzyme active site would still be targets for second, humanised and galactosylated antibodies or alternatively the genetically engineered molecules could be optimized to achieve both tumour localisation and plasma clearance.

It isn't possible to predict how far genetic engineering will be able to achieve these macromolecular reconstructions but no insurmountable problems have yet been defined so we may continue to live hopefully. This is not to suggest that the ADEPT approach has no limitations. Antibody uptake in relative terms tends to diminish as tumours enlarge (Pedley *et al.*, 1987), so we may have doubts about its effectiveness against large tumour masses. There is also the question of whether the blood/brain barrier can be circumvented. (Begent, 1985; Richardson *et al.*, 1986). Local accumulations of enzyme activity could be a limiting factor through immunoconjugate deposition at non-tumour sites. In the choriocarcinoma model both radiolabelled anti-hCG (Searle *et al.*, 1981) and anti-hCG-enzyme conjugates have shown high levels in lung tissue, probably due to accumulation in lung macrophages. Lung damage and fibrosis would be potentially serious limiting factors. A further concern is the marker-less cell with clonogenic potential in isolation at sites distant from marker expressing cells. Clearly, such cells are likely to escape any antibody based attack which provides cytotoxic effects for only a few hours. Perhaps those cells will be susceptible if the cytotoxic effect is sustained for several days or if it is delivered repeatedly, but here I am plunging into deep speculation. Studies with metastasising tumour models may show how important this could be and whether effective strategies can be evolved.

However, there is no reason on present evidence to close on a pessimistic note. A wide repertoire of monoclonal antibodies with interesting specificities continues to emerge. Prodrugs from which can be generated active drugs several orders of magnitude more potent than those we have so far tested will be synthesised. The development of matching pairs of enzymes and prodrugs with improved characteristics presents a considerable but not an unrealistic challenge and it seems possible that a whole new field of therapy will emerge.

I wish to thank the Cancer Research Campaign for its support and all the Medical Oncology team at Charing Cross. Those contributing to the ADEPT programme include: P. Antoniw, J. and R. Boden, G. Boxer, P. Burke, A. Green, B. Pedley, D. Read, G. Rogers, F. Searle, S. Sharma, C. Springer. I also thank R. Sherwood and R. Melton, Public Health Laboratory Service, Porton; M. Jarman, Institute of Cancer Research, Sutton and Dr. T. Connors MRC Toxicology Unit, Carshalton for much valued collaboration.

### References

ABUCHOWSKI, A., DAVIS, F.F. & DAVIS, S. (1981). Immunosuppressive properties and circulating life of achromobacter Glutaminase-Asparaginase covalently attached to polyethylene glycol in man. *Cancer Treatment Rep.*, **65**, 1077.

BAGSHAWE, K.D. (1983). Tumour markers – where do we go from here? *Br. J. Cancer*, **48**, 167.

BAGSHAWE, K.D. (1987). Antibody directed enzymes revive anticancer prodrugs concept. *Br. J. Cancer*, **56**, 531.

BAGSHAWE, K.D., SPRINGER, C.J., SEARLE, F. *et al.* (1988). A cytotoxic agent can be generated selectively at cancer sites. *Br. J. Cancer*, **58**, 700.

BEGENT, R.H.J., SEARLE, F., STANWAY, G. *et al.* (1981). Radioimmunolocalisation of tumours by external scintigraphy after administration of $^{131}$I antibody to human chorionic gonadotrophin: preliminary communication. *J. R. Soc. Med.*, **73**, 624.

BEGENT, R.H.J., GREEN, A.J., BAGSHAWE, K.D. *et al.* (1982). Liposomally entrapped second antibody improves tumour imaging with radiolabelled (first) antitumour antibody. *Lancet*, **ii**, 739.

BEGENT, R.H.J. (1985). Radioimmunolocalisation of cerebral tumours. *Prog. Exp. Tumour Res.*, **29**, 78.

BOULIANNE, G.L., ISENMAN, D.E., HOZUMI, N. & SHULMAN, M.J. (1987). Biological properties of chimeric antibodies. *Mol. Biol. Med.*, **4**, 37.

BROWNE, P.J. & BAGSHAWE, K.D. (1982). Enhancement of human chorionic gonadotrophin production by antimetabolites. *Br. J. Cancer*, **46**, 22.

BUCHEGGER, F., VACCA, A., CARREL, S. *et al.* (1988). Radioimmunotherapy of human colon carcinoma by $^{131}$I-labelled monoclonal anti-CEA antibodies in a nude mouse model. *Int. J. Cancer*, **41**, 127.

CAPONE, P.M., PAPSIDERO, L.D. & CHU, T.M. (1984). Relationship between antigen density and immunotherapeutic response elicited by monoclonal antibodies against solid tumours. *J. Natl Cancer Inst.*, **72**(3), 673.

CHAKRABARTY, S., TOBON, A., VARANI, J. & BRATTAIN, M. (1988). Induction of carcinoembryonic antigen secretion and modulation of protein secretion/expression and fibronectin/laminin expression in human colon carcinoma cells by transforming growth factor-$\beta^1$. *Cancer Res.*, **48**, 4059.

CHOU, J.Y., ROBINSON, J.C. & WANG, S.S. (1977). Effects of sodium butyrate on synthesis of human chorionic gonadotrophin in trophoblastic and non-trophoblastic tumours. *Nature*, **268**, 543.

CHOU, J.Y. & ROBINSON, J.C. (1977). Induction of placental alkaline phosphatase in choriocarcinoma cells by 5-bromo-2'-deoxyuridine. *In Vitro*, **13**, 450.

CLARK, M.R. & WALDMANN, H. (1987). T-cell killing of target cells induced by hybrid antibodies: comparison of two bispecific monoclonal antibodies. *J. Natl Cancer Inst.*, **79**, 1393.

COBB, L.M. (1967). Chemotherapeutic activity and pathological effects of an alkylataing agent of short half-life designed for intra-arterial infusion (CB1850). *Int. J. Cancer*, **2**, 5.

DAIRKEE, S.H. & HACKETT, A.J. (1988). Internal antigens accessible in breast cancer: implications for tumor targeting. *J. Natl Cancer Inst.*, **80**(15), 1216.

DEGUCHI, T., CHU, T., LEONG, S.S. *et al.* (1986). Effect of methotrexate-monoclonal anti-prostatic acid phosphatase antibody conjugate on human prostate tumour. *Cancer Res.*, **46**, 3751.

DURRANT, L.G., ROBINS, A., MARKMAN, R.A. *et al.* (1989). Abrogation of antibody responses in rates to murine monoclonal antibody 719T/36 by treatment with daunomycin-cis-aconityl-719T/36 conjugates. *Cancer Immunol. Immunother.* (in press).

DYKES, P.W., BRADWELL, A.R., CHAPMAN, C.E. & VAUGHAN, A.T.M. (1987). Radioimmunotherapy of cancer: clinical studies and limiting factors. *Cancer Treatment Rev.*, **14**, 87.

EBINA, T., SATAKE, M. & ISHIDA, N. (1977). Inhibition of surface immunoglobulin central capping of Daudi cells and cell spreading of HeLa-S3 cells by Neocarzinostatin. *Cancer Res.*, **37**, 4423.

ENDO, Y. & TSURIGI, K. (1987). RNA N-glycosidase activity of Ricin A Chain. *J. Biol. Chem.*, **262**, 8128.

FREI, E., TEICHER, B.A., HOLDEN, S.A. *et al.* (1988). Preclinical studies and clinical correlation of the effect of alkylating dose. *Cancer Res.*, **48**, 6417.

GHOSE, T. & NIGAM, S.P. (1972). Antibody as carrier of chlorambucil. *Cancer*, **29**, 1398.

GLENNIE, M.J. & STEVENSON, G.T. (1982). Univalent antibodies kill tumour cells *in vitro* and *in vivo*. *Nature*, **295**, 712.

GLENNIE, M.J. BRENNAND, D.M., BRYDEN, F. *et al.* (1988). Bispecific F(ab')$_2$ antibody for the delivery of Saporin in the treatment of lymphoma. *J. Immunol.* **141**, 3662.

GUADAGNI, F., SCHLOM, J., POTHEN, S. *et al.* (1988). Parameters involved in the enhancement of monoclonal antibody targeting *in vivo* with recombinant Interferon. *Cancer Immunol. Immunother.*, **26**, 222.

HALE, G., SWIRSKY, D.M., HAYHOE, F.G.J. & WALDMANN, H. (1983). Effects of monoclonal anti-lymphocyte antibodies *in vivo* in monkeys and humans. *Mol. Biol. Med.*, **1**, 321.

HATTNER, R.S., HUBERTY, J.P., ENGELSLAD, B.L. *et al.* (1984). Localisation of m-iodo (I$^{131}$) benzylguamidine in neuroblastoma. *Am. J. Roentgenol.*, **143**, 373.

HOSHINA, M., HUSSA, R., PATTILLO, R. & BOIME, I. (1983). Cytological distribution of chorionic gonadotrophin subunit and placental lactogen messenger RNA in neoplasms derived from human placenta. *J. Cell Biol.*, **97**, 1200.

JAIN, R.K. & BAXTER, L.T. (1988). Mechanisms of heterogeneous distribution of monoclonal antibodies and other macromolecules in tumors: significance of elevated interstitial pressure. *Cancer Res.*, **48**, 7022.

KANNELOS, J., PIETERSZ, G.A. & McKENZIE, I.F.C. (1985). Studies of methotrexate-monoclonal antibody conjugates for immunotherapy. *J. Natl Cancer Inst.*, **75**, 319.

KLIBANOFF, A.L., MARTYNOV, A.V., SLINKIN, M.A. *et al.* (1988). Blood clearance of radiolabelled antibody: Enhancement by lacto-samination and treatment with biotin-avidin or anti-mouse IgG antibodies. *J. Nucl. Med.*, **29**, 1951.

KRENNING, E.P., BREEMAN, W.A.P., KOOIJ, P.P.M. *et al.* (1989). Localisation of endocrine relating tumour with radioiodinated analogue of somatostatin. *Lancet*, **i**, 242.

LEDERMANN, J.A., BEGENT, R.H.J. & BAGSHAWE, K.D. (1988a). Cyclosporin A prevents the anti-murine antibody response to a monoclonal anti-tumour antibody in rabbits. *Br. J. Cancer*, **58**, 562.

LEDERMANN, J.A., BEGENT, R.H.J., BAGSHAWE, K.D. *et al.* (1988b). Repeated antitumour antibody therapy in man with suppression of the host response to Cyclosporin A. *Br. J. Cancer*, **58**, 654.

LERNER, R.A. & TRAMONTANO, A. (1987). Antibodies as enzymes. *TIBS*, **12**, 427.

LEWIS, J.C.M., SMITH, P.A., KEEP, P.A. & BOXER, G.M. (1983). A comparison of the content and immunohistochemical patterns of CEA-like activity in human colorectal tumours and nude mouse xenografts. *Exp. Pathol.*, **24**, 227.

MASON, D.W. & WILLIAMS, A.F. (1980). The kinetics of antibody binding to membrane antigens in solution and at the cell surface. *Biochem. J.*, **187**, 1.

MORRISON, S.L., JOHNSON, M.J., HERZENBERG, L.A. & OI, V.T. (1984). Chimeric human antibody molecules: Mouse antigen-binding domains with human constant region domains. *Proc. Natl Acad. Sci.*, **81**, 6851.

MORRISON, S.L., CANFIELD, S., PORTER, S. *et al.* (1988). Production and characterisation of genetically engineered antibody molecules. *Clin. Chem.*, **34**, 1668.

NEUBERGER, M.S., WILLIAMS, G.T. & FOX, R.O. 1984). Recombinant antibodies possessing novel effector functions. *Nature*, **312**, 604.

ORLANDI, R., CANEVARI, S., LEONI, F. *et al.* (1986). Change in binding reactivity of an anti-tumor monoclonal antibody after introduction of 2-pyridyl disulphide groups. *Hybridoma*, **5**, 1.

PEREZ, P., HOFFMAN, R.W., SHAW, S. *et al.* (1985). Specific targeting of cytotoxic T-cells by anti-T3 linked to anti-target cell therapy. *Nature*, **316**, 354.

POLLACK, S.J., JACOBS, J.W. & SCHULTZ, P.G. (1986). Selective chemical catalysis by an antibody. *Science*, **234**, 1570.

PRIMUS, F.J., KUHNS, W.J. & GOLDENBERG, D.M. (1983). Immunological heterogeneity of carcinoembryonic antigen: immunohistochemical detection of carcinoembryonic antigen determinants in colonic tumors with monoclonal antibodies. *Cancer Res.*, **44**, 693.

REICHMANN, L., FOOTE, J. & WINTER, G. (1988a). Expression of an antibody Fv fragment in myeloma cells. *J. Mol. Biol.*, **203**, 825.

REICHMANN, L., CLARK, M., WALDMANN, H. & WINTER G. (1988b). Reshaping human antibodies for therapy. *Nature*, **332**, 324.

RICHARDSON, R.B., DAVIES, A.G., BOURNE, S.P. *et al.* (1986). Radioimmunolocalisation of human brain tumours: biodistribution of radiolabelled monoclonal antibody UJ131A. *Eur. J. Nucl. Med.*, **12**, 313.

ROWE, R.E., PIMM, M.V. & BALDWIN, R.W. (1985). Anti-idiotype antibody responses in cancer patients receiving a murine monoclonal antibody. *ICRS Med. Sci.*, **13**, 936.

SEARLE, F., BODEN, J., LEWIS, J.C.M. *et al.* (1981). A human choriocarcinoma xenograft in nude mice: a model for the study of antibody localisation. *Br. J. Cancer*, **44**, 137.

SEARLE, F., BIER, C., BUCKLEY, R.G. *et al.* (1986). The potential of carboxypeptidase G2 antibody conjugates as anti-tumour agents. 1. Preparation of anti human chorionic gonadotrophin-carboxypeptides G2 and cytotoxicity of the conjugate against JAR choriocarcinoma cells *in vitro*. *Br. J. Cancer*, **53**, 377.

SENTER, P.D., SAULNIER, M.G., SCHREIBER, G.J. *et al.* (1988). Antitumor effects of antibody-alkaline phosphatase conjugates in combination with etoposide phosphate. *Proc. Natl Acad. Sci. USA*, **85**, 4842.

SHARKEY, R.M., KALTOVICH, F.A., SHIH, L.B. *et al.* (1988). Radioimmunotherapy of human colonic cancer xenografts with $^{90}$Y-labelled monoclonal antibodies to carcinoembryonic antigen. *Cancer Res.*, **48**, 3270.

SHOUVAL, D., SHAFRITZ, D.A., ZURAWSKI, V.R. *et al.* (1982). Immunotherapy in nude mice of human hepatoma using monoclonal antibodies against Hepatitis B virus. *Nature*, **298**, 567.

STAERZ, U.D. & BEVAN, M.J. (1986). Hybrid hybridoma producing a bispecific monoclonal antibody that can focus effector T-cell activity. *Proc. Natl Acad. Sci., USA*, **83**, 1453.

STEVENSON, G.T. & STEVENSON, F.K. (1975). Antibody to a molecularly defined antigen confined to a tumour cell surface. *Nature*, **254**, 714.

SUTHERLAND, R., BUCHEGGER, F., SCHREYER, M. *et al.* (1987). Penetration and binding of radiolabelled anti-carcinoembryonic antigen monoclonal antibodies and their antigen binding fragments in human colon multicellular tumor spheroids. *Cancer Res.*, **47**, 1627.

TAYLOR, R.B., DUFFUS, P.H., RAFF, M.C., DE PETRIS, S. (1971). Redistribution and pinocytosis of lymphocyte surface immunoglobulin molecules induced by anti-immunoglobulin antibody. *Nature New Biol.*, **233**, 225.

THORPE, P.E., ROSS, W.C.J., CUMBER, A.J. *et al.* (1978). Toxicity of diphtheria toxin for lymphoblastoid cells is increased by conjugation to antilymphocytic globulin. *Nature*, **271**, 752.

WALDMANN, H., HALE, G., CLARK, M. *et al.* (1988). Monoclonal antibodies for immunosuppression. *Prog. Allergy*, **45**, 16.

WILKINSON, I., JACKSON, G.J.C., LANG, G.M. *et al.* (1987). Tolerance induction in mice by conjugates of monoclonal immunoglobulins and monomethoxypolyethylene glycol. *J. Immunol.*, **139**, 326.

WILSON, E.A. & JAWAD, M.J. (1982). Stimulation of human chorionic gonadotrophin secretion by glucocorticoids. *Am. J. Obstet. Gynecol.* **142**, 344.